# Antibacterial activity and safety of commercial veterinary cationic steroid antibiotics and neutral superoxidized water

**DOI:** 10.1371/journal.pone.0193217

**Published:** 2018-03-07

**Authors:** Benjamin E. Bergstrom, Ahmed Abdelkhalek, Waleed Younis, G. Kenitra Hammac, Wendy M. Townsend, Mohamed N. Seleem

**Affiliations:** 1 Department of Veterinary Clinical Sciences, College of Veterinary Medicine, Purdue University, West Lafayette, Indiana, United States of America; 2 Department of Comparative Pathobiology, College of Veterinary Medicine, Purdue University, West Lafayette, Indiana, United States of America; Bharathidasan University, INDIA

## Abstract

Antibiotic resistance of bacteria common to the ocular surface is an evolving problem. Thus, novel treatment options with new modes of action are required. We investigated the antibacterial activity and safety of three commercially available topical veterinary ophthalmic products (cationic steroid antibiotics, products A and B, and a neutral superoxidized water, product C) to determine their potential use as antimicrobial alternatives. The minimum inhibitory concentrations (MIC) of the three products were determined against 17 antibiotic resistant bacterial clinical isolates from the ocular surface. Using a standard cytotoxicity assay, the products at varying concentrations were evaluated with a corneal fibroblast cell line and a macrophage-like cell line to determine their potential toxic effect in vitro. The commercial ophthalmic solutions, ofloxacin 0.3%, tobramycin 0.3% and gentamicin 0.3% were used as positive controls for the MIC and tobramycin 0.3% was used as positive control for the cytotoxicity assays. For the MIC, Product C showed no inhibition of growth for any organisms, while Products A and B showed inhibition of growth similar to slightly less than the positive controls. For the cytotoxicity assays, Product C exhibited minimal toxicity while Products A and B exhibited toxicity similar to the controls. In conclusion, Product C had no antibacterial activity in these assays, while Products A and B had antibacterial profiles similar to slightly less than common topical ophthalmic antibiotics and cytotoxicity profiles similar to common topical ophthalmic antibiotics. To our knowledge, this is the first report on the antibacterial activity and safety of the cationic steroid antibiotics and superoxidized water.

## Introduction

Multi-drug resistant organisms of the corneal and conjunctival surfaces are a growing concern in numerous veterinary species [1–5]. Recently, a study investigating the antimicrobial resistance of 49 isolates of *Staphylococcus pseudintermedius* from the eyes of dogs with and without ocular disease concluded that fluoroquinolone resistance was increasing. Eighteen of 49 isolates were *mec-A* positive, 16 of which were also oxacillin resistant. Nine of the mec-A positive were resistant to more than one fluoroquinolone [1]. In another study investigating the prevalence of MRSA-associated keratitis in dogs, 71 *Staphylococcus* strains were isolated. Twenty-four percent of those strains were methicillin resistant [2]. The increasing prevalence of multi-drug resistant bacteria illustrates the need for antimicrobial agents targeting novel sites to circumvent resistance.

One appealing target is the bacterial membrane since resistance to membrane-targeting antibiotics would require major changes in bacterial structure. The absence of the target in the mammalian genome is also desirable because bacterial membrane-targeting antimicrobials should therefore not interfere with essential mammalian functions [6–9].

Antimicrobial peptides (AMPs) target the bacterial cell membrane and are produced by the innate immune system. AMPs typically consist of a short chain of 12 to 100 amino acids linked by peptide bonds [10]. They are most often cationic (with at least two positive charges) which permits their interaction with negatively charged components present in the bacterial membrane and cell wall. AMPs are also amphipathic, which allows them to target and penetrate bacterial cell membranes [11–15]. Since their discovery, over 2700 antimicrobial peptides have been isolated. Unfortunately, despite the success in isolating AMPs, they are very sensitive to temperature, expensive to manufacture in bulk, and selecting appropriate packaging material and storage conditions to enhance shelf life and stability is an ongoing struggle [11, 16].

Similar to AMPs, some peptide antibiotics such as polymyxin B, also target the bacterial membrane. Polymyxin B has been characterized as selectively binding lipid A, the primary constituent of the outer membranes of Gram-negative bacteria, and this peptide antibiotic is highly active against most strains of bacteria producing lipid A [17–20]. Polymyxin B is also considered facially amphipathic, meaning it contains side chains from hydrophobic residues on one face of a molecule and cationic side chains on the opposing face [7, 21]. In recent years, a class of cationic steroid antibiotics (CSAs) was developed with the intent of mimicking the antibacterial activities of polymyxin B [17, 22]. These compounds, prepared from cholic acid with carbohydrates appended on one face of the molecule, were shown to facilitate the movement of polar compounds across eukaryotic membranes [23]. Since their development, several studies have evaluated various CSAs to determine the antibacterial efficacies of the individual compounds. One CSA in particular, CSA-13, has been shown to have potent broad-spectrum antimicrobial activity [10, 24–31]. Where AMPs have fallen short in their capacity for large-scale production, CSAs have succeeded. CSA-13 has been manufactured into formulations for commercial veterinary use and is currently marketed as Products A and B. The concentration of the active CSA-13 in these products is 0.04%. Although marketed under different names, the products appear to be the same formulations as determined by a personal communication with the product developer.

Another antimicrobial alternative currently being investigated is neutral superoxidized water (SOW). Super-oxidized waters have historically been used as disinfectants in hospitals and are produced as either acidic (pH 2–4) or alkaline (pH ≥ 9) solutions [32–35]. Unfortunately, acidic and alkaline SOWs are very corrosive, unstable due to very short half-lives, and unsafe for medicinal and therapeutic use [32, 36]. More stable, neutral SOWs have therefore been investigated as antimicrobials and wound irrigants due to their improved tissue compatibility [32, 35, 37–43]. Neutral SOWs are electrochemically processed aqueous solutions manufactured from pure water and salt. Electrical energy is used to produce a charge in the aqueous solution resulting in a solution rich in reactive oxygen species, a neutral pH, and a longer half-life (> 12 months) than other super-oxidized solutions such as bleach [38, 44]. The chemically active oxychlorine compounds in neutral SOW, hypochlorous acid and sodium hypochlorite, exist in equilibrium to produce solutions with high efficacy against microorganisms while maintaining product stability and low toxicity [32]. The mechanism by which SOWs are antimicrobial has not been fully defined, but is thought to be similar to the respiratory burst phenomenon displayed by phagocytic leukocytes. Briefly, when bacteria are engulfed by neutrophils, the neutrophils rapidly produce and release reactive oxygen species that lead to the formation of hypochlorous acid. Hypochlorous acid is the main active ingredient in neutral SOW. The neutral charge of the hypochlorous acid molecules draw them to the positively charged bacterial cell membrane. The hypochlorous acid molecules penetrate the membrane, enter the cell, and begin disrupting the normal metabolic functions of the bacteria. Cell lysis occurs via the osmotic pressure imbalance between the cell and the hypotonic SOW solution. Using contrast microscopy, bacterial swelling was observed within the first 30 seconds of exposure to SOW followed by bacterial cell rupture [45]. One such neutral SOW, commercially manufactured for the veterinary market, is Product C. The total free available chlorine in the commercial solution ranges from 110 to 150 parts per million (ppm) and the main active ingredient, hypochlorous acid, is present at a concentration of 0.015%.

To our knowledge, there are no data on the antibacterial activity and safety of the CSAs, Products A and B, and the neutral SOW, Product C, as their commercial, over the counter, veterinary formulations. These products are not animal drugs approved by the FDA. Instead they are considered to be homeopathic due to the very low concentrations of active ingredients. Homeopathic products are minimally regulated by the FDA and there is no requirement to demonstrate efficacy, lack of toxicity, or stability.

In the present study, we investigated the antibacterial activity of these three commercial, over the counter, veterinary formulations against clinical bacterial isolates and the toxicity in two different mammalian cell lines. If proven safe and effective, their use as antimicrobial alternatives could be further investigated in the efforts to limit antibiotic use and combat antimicrobial resistance.

## Materials and methods

### Commercial products, antibiotics and reagents

The cationic steroid antibiotics, Products A and B, (both products contain cholan-24-oic acid, 3,7,12-tris[3-amino-1-oxopropoxy]-octyl ester [3 alpha,5 beta,7 alpha,12 alpha]-[CSA-13 0.04%], polysorbate 20, hydroxypropyl methylcellulose, water); neutral superoxidized water, Product C (contains hypochlorous acid [0.0150%], electrolyzed water, sodium chloride, phosphates, sodium hypochlorite); and ophthalmic antibiotic solutions, ofloxacin 0.3%, (Akorn Inc., Lake Forest, IL, USA), tobramycin 0.3% (Bausch & Lomb Inc., Tampa, FL, USA) and gentamicin 0.3% (Bausch & Lomb Inc., Tampa, FL, USA), were all purchased from commercial vendors. Mueller-Hinton broth (MHB) was purchased from Sigma-Aldrich.

### Bacterial isolates

Seventeen total clinical bacterial isolates of different bacterial strains (4 *Moraxella* spp., 1 *Neisseria* spp., 3 *Pseudomonas* spp., 5 *Staphylococcus* spp. and 4 *Streptococcus* spp.) were included in this study. All isolates, except the *Moraxella* spp., were identified at the Indiana Animal Disease Diagnostic Laboratory from specimens collected from canines admitted to the Purdue University Veterinary Teaching Hospital. All canine isolates were considered multi-drug resistant. The *Moraxella* spp. isolates were obtained and identified at the Veterinary Diagnostic Center at the University of Nebraska from infectious bovine keratoconjunctivitis. Briefly, swabs were streaked onto tryptic soy agar containing 5% sheep’s blood and incubated for 18–24 hr at 37°C with 5% CO2 supplementation. Bacterial colonies with morphology consistent with members of the genus Moraxella were further screened for oxidase production. Colonies positive for oxidase production were then subjected to Gram staining and were subcultured for purity. All subcultured organisms that were characterized as Gram-negative rods or coccobacilli by Gram stain were subjected to molecular speciation. Isolates were subsequently identified as M. bovis based on a polymerase chain reaction (PCR) assay and a subsequent restriction fragment length polymorphism analysis that targets the 16S-23S intergenic spacer region in combination with phenotypic tests as needed [46]. A description of the clinical isolates used in the study can be found in the Supplemental Table (S1 Table).

### Antibacterial assays

The broth microdilution technique was used to determine the minimum inhibitory concentrations (MIC) of the commercial products and antibiotics in Mueller Hinton Broth (MHB)in accordance with the Clinical and Laboratory Standards Institute (CLSI) guidelines with few modifications [47]. MIC assays were carried out with an initial bacterial inoculum of 5 x 10^5^ colony forming unit (CFU/ml). Commercial products and antibiotics were added to Polysterene 96-well plates (Corning, Corning, NY, USA) different concentrations, serially diluted and plates were incubated for 24 hr. at 37°C. MIC was defined as the lowest concentration of commercial product or antibiotic that inhibited the visible growth of bacteria. MIC was done in triplicate and the highest value was reported. To standardize results, minimum inhibitory concentrations were calculated in μg/ml from the original dilutions of each of the original commercial products because the starting concentrations of the active ingredients in each of the products were different from one another. For example, the commercial antibiotics (Tobramycin, Gentamicin, Ofloxacin) have a 0.3% concentration of their respective antibiotic whereas the cationic steroid antibiotics (Products A and B) have a 0.04% concentration of CSA-13 and Product C has a 0.015% concentration of hypochlorous acid. For the *Moraxella* and *Streptococcus* spp. strains, 5% sheep’s blood was added to the growth media.

### Cytotoxicity analyses

Commercial products were assayed at concentrations of 6.25%, 12.5%, 25%, 50% and 100% against a murine macrophage-like cell line (J774A.1) and a rabbit corneal fibroblast cell line (CCL-60) to determine the potential toxic effects in vitro. The J774A.1 line was used to evaluate possible effects of the topical medications if they are absorbed systemically and the CCL-60 line was used to evaluate direct effects on the corneal surface. Briefly, J774A.1 cells were seeded at a density of 1.5 x 10^4^ per well in a tissue culture treated 96-well plate (Corning, Corning, NY, USA) in Dulbecco’s Modified Eagle Medium (DMEM) supplemented with 10% fetal bovine serum (FBS), and incubated at 37°C in a 5% CO_2_ atmosphere for 24 hours. Similarly, the CCL-60 cells were seeded at a density of ~2 x 10^4^ cells per well in a tissue culture treated 96-well plate, also suspended in DMEM supplemented with 10% FBS and incubated at 37°C in a 5% CO_2_ atmosphere for 24 hours. The compounds were added in triplicate and incubated at differing intervals for different assays, a single 2-hour interval and four 5-minute intervals. For the multiple exposures, the cells were washed, new media was added, and the cells were incubated for 1 hour before re-addition of the compound. The 5-minute intervals were used to simulate the amount of time a drop would likely remain on the surface of the eye before being washed away by the tear film. The multiple time points were used to simulate the number of times per day an eye drop would likely be used in a clinical setting. The cells were treated with commercial products at concentrations ranging from their initial marketed concentration to 0.8% of the initial concentration. Untreated cells were used as a negative control. Tobramycin 0.3% ophthalmic solution was used as a positive control. After incubation, the cells were washed three times with PBS and the media in each well was replaced with 100 μL of DMEM media and 20% of the assay reagent, MTS 3-(4,5-dimethylthiazol-2-yl)-5-(3-carboxymethoxyphenyl)-2-(4-sulfophenyl)-2*H*-tetrazolium) (Promega, Madison, WI, USA). The cells were incubated further for 4 hours at 37°C in 5% CO_2_. Corrected absorbance readings were taken using a kinetic ELISA microplate reader (Molecular Devices, Sunnyvale, CA, USA). Cell viability was expressed as percentage of absorbance in comparison to the control, PBS.

### Statistical analyses

Cytotoxicity data were analyzed using two-way ANOVA with Holm-Sidak’s multiple comparisons test using GraphPad Prism 6.0 (GraphPad Software, La Jolla, CA, USA). P-values of < 0.05 were considered significant.

## Results

### Antibacterial assays

The bacterial species and MICs for each of the compounds are shown in Table 1. For the *Moraxella* spp., Products A and B showed similar, moderate inhibitory activity. Product C caused no inhibition of growth. The commercial antibiotics, however, showed potent inhibitory activity against all of the *Moraxella* spp. isolates. For the single *Neisseria* sp. isolate, the CSAs were less effective than the commercial antibiotics while Product C showed no inhibition of growth. For the *Pseudomonas* spp., the CSAs and the commercial antibiotics demonstrated potent inhibitory activity. Product C, showed no inhibition of growth of any of the *Pseudomonas* organisms. For the *Staphylococcus* spp., both Products A and B showed inhibitory activity similar to the commercial antibiotics, which demonstrated moderate inhibitory activity. Product C showed no inhibition of growth to any of the *Staphylococcus* organisms at the concentration tested. For the *Streptococcus* organisms both CSAs demonstrated moderate inhibitory activity compared to the commercial antibiotics, which showed potent inhibitory activity. Product C showed no inhibition of growth for any of the *Streptococcus* organisms.

**Table 1:** Minimum inhibitory concentrations (μg/ml) of Products A, B, C and commercial topical ophthalmic products against bacterial isolates.

	MICs of the tested preparation in μg/ml.					
Strain	A	B	C	Tobramycin	Gentamicin	Ofloxacin
*Moraxella bovis* 80–2013000742	0.78	≥ 6.25	≥ 1500	≤ 0.36	≤ 0.36	≤ 0.36
*Moraxella bovis* 36–2012001922	6.25	6.25	≥ 1500	≤ 0.36	≤ 0.36	≤ 0.36
*Moraxella bovis* 120–2014002878	6.25	≥ 6.25	≥ 1500	≤ 0.36	≤ 0.36	≤ 0.36
*Moraxella bovis* 42–2012003587	6.25	≥ 6.25	≥ 1500	≤ 0.36	≤ 0.36	≤ 0.36
*Neisseria spp*. isolate 1	6.25	6.25	≥ 1500	≤ 11.7	≤ 11.7	≤ 11.7
*Pseudomonas aeruginosa* 15442	6.25	6.25	≥ 1500	≤ 0.36	0.73	0.73
*Pseudomonas aeruginosa* isolate 1	6.25	6.25	≥ 1500	≤ 0.36	≤ 0.36	≤ 0.36
*Pseudomonas aeruginosa* isolate 2	25	6.25	≥ 1500	≤ 0.36	≤ 0.36	5.8
*Staphylococcus aureus* isolate 1	6.25	6.25	≥ 1500	≤ 0.36	≤ 0.36	≤ 0.36
*Staphylococcus aureus* isolate 2	6.25	6.25	≥ 1500	0.73	0.73	≤ 0.36
*Staphylococcus pseudintermedius* Case one	3.13	6.25	≥ 1500	11.7	23.4	23.4
*Staphylococcus pseudintermedius* Case two	3.13	3.13	≥ 1500	≤ 11.7	≤ 0.391	≤ 0.391
*Streptococcus canis* isolate 1	12.5	≥ 6.25	≥ 1500	5.8	2.9	0.73
*Streptococcus canis* isolate 2	12.5	≥ 6.25	≥ 1500	11.7	5.8	0.73
*Streptococcus canis* isolate 3	12.5	6.25	≥ 1500	5.9	2.9	1.5
*Streptococcus canis* isolate 4	6.25	≥ 6.25	≥ 1500	11.7	5.8	0.73
*Streptococcus schleiferi* isolate 1	0.78	≥ 6.25	≥ 1500	≤ 0.36	≤ 0.36	≤ 0.36

All MIC values are recorded in μg/ml. Concentrations were calculated from the dilutions of the active ingredient in each of the original commercially available products.

### Cytotoxicity analyses

**Fig 1:**
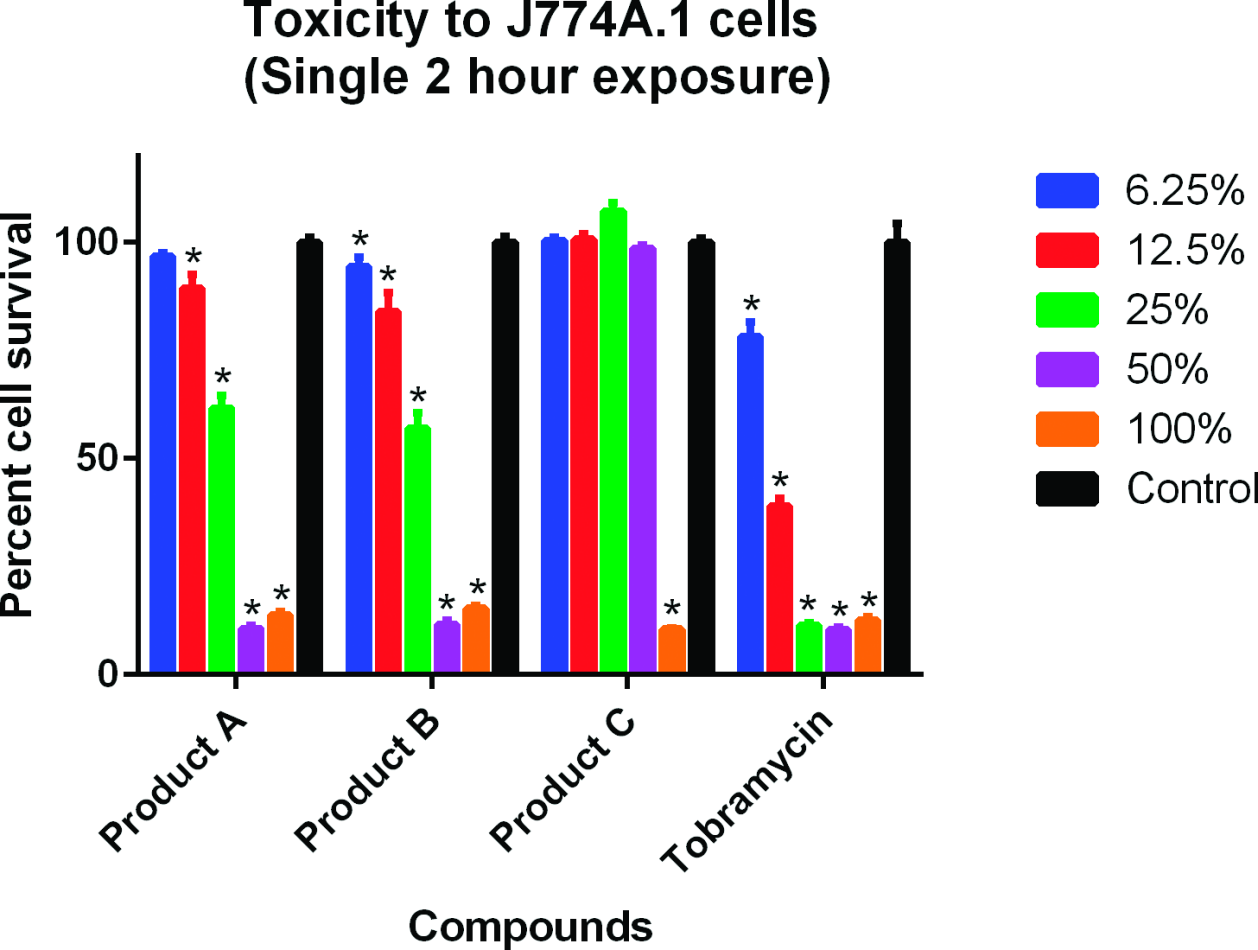
Cytotoxicity of ophthalmic products against J774A.1 cells, single 2-hour exposure. Tobramycin was used as the positive control. Cells were exposed to the commercial products for a single 2-hour exposure. Cell viability was expressed as percentage of absorbance in comparison to the negative control. A compound was considered toxic to the cell line in this experiment if ≤ 95% cell survival was recorded. Toxicity is indicated by (*).

**Fig 2:**
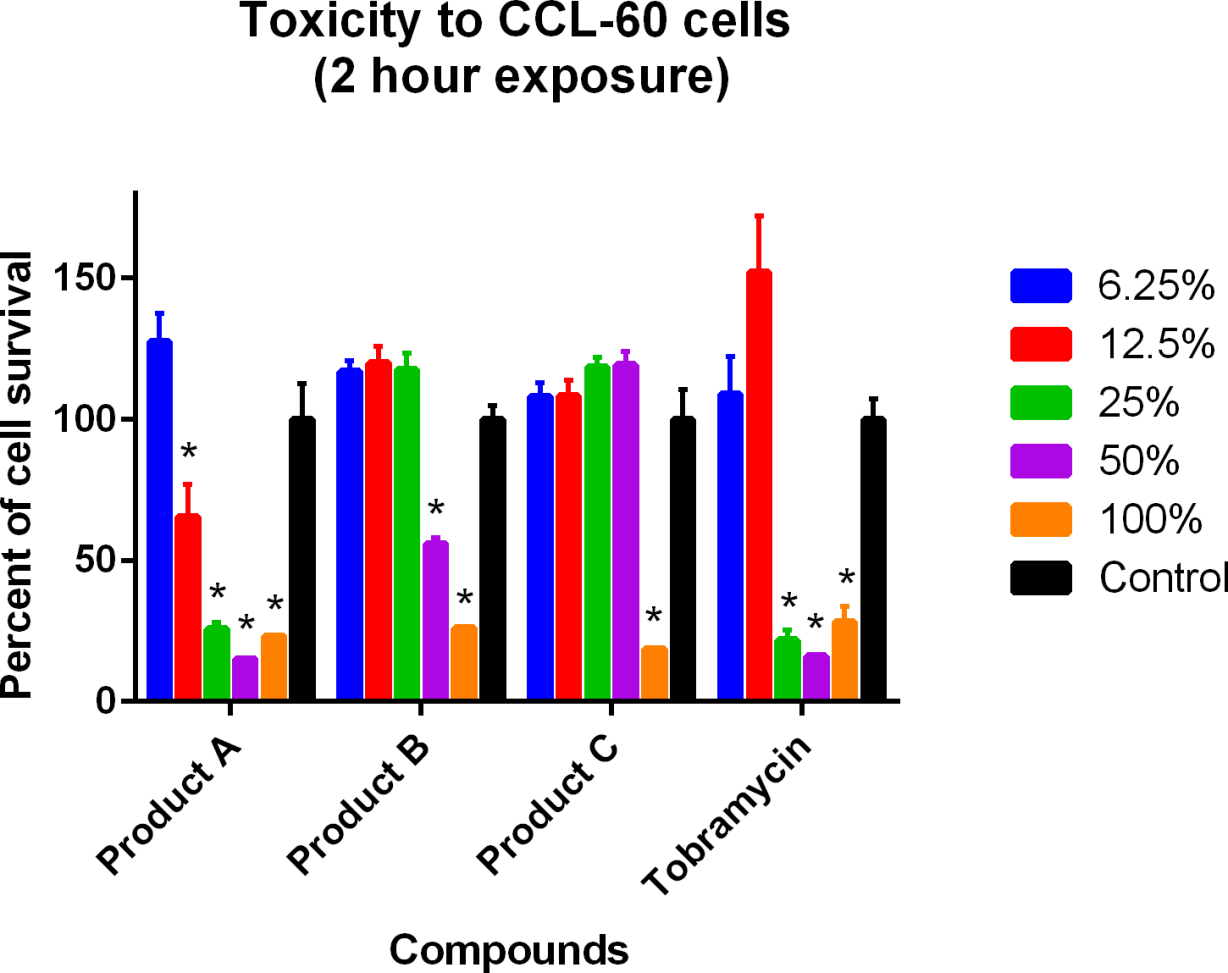
Cytotoxicity of ophthalmic products against CCL-60 cells, single 2-hour exposure. Tobramycin was used as the positive control. Cells were exposed to the commercial products for a single 2-hour exposure. Cell viability was expressed as percentage of absorbance in comparison to the negative control. A compound was considered toxic to the cell line in this experiment if ≤ 95% cell survival was recorded. Toxicity is indicated by (*).

**Fig 3:**
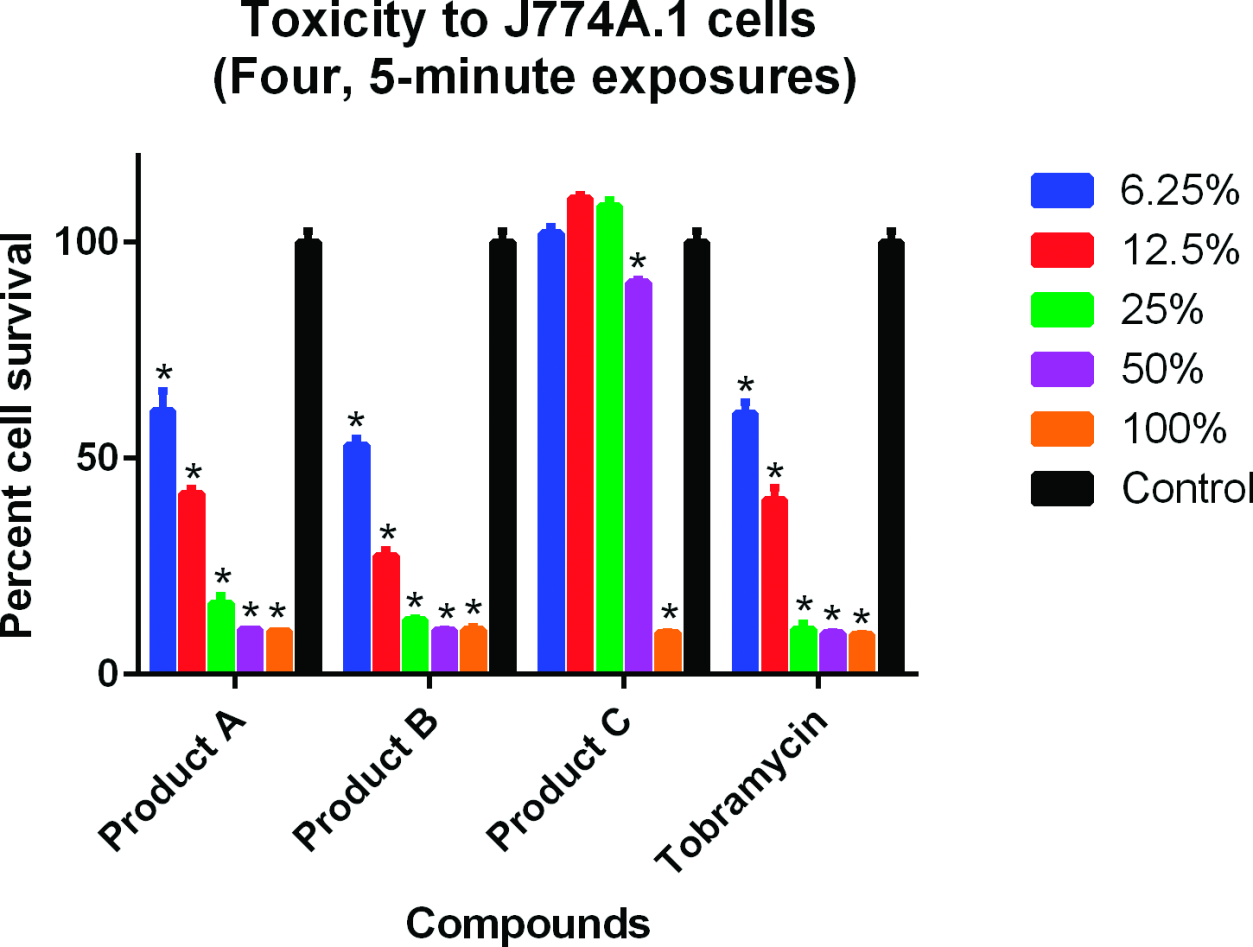
Cytotoxicity of ophthalmic products against J774A.1 cells, four 5-minute exposures. Tobramycin was used as the positive control. Cells were exposed to the commercial products for 5 minutes, 4 separate times. Cell viability was expressed as percentage of absorbance in comparison to the negative control. A compound was considered toxic to the cell line in this experiment if ≤ 95% cell survival was recorded. Toxicity is indicated by (*).

**Fig 4:**
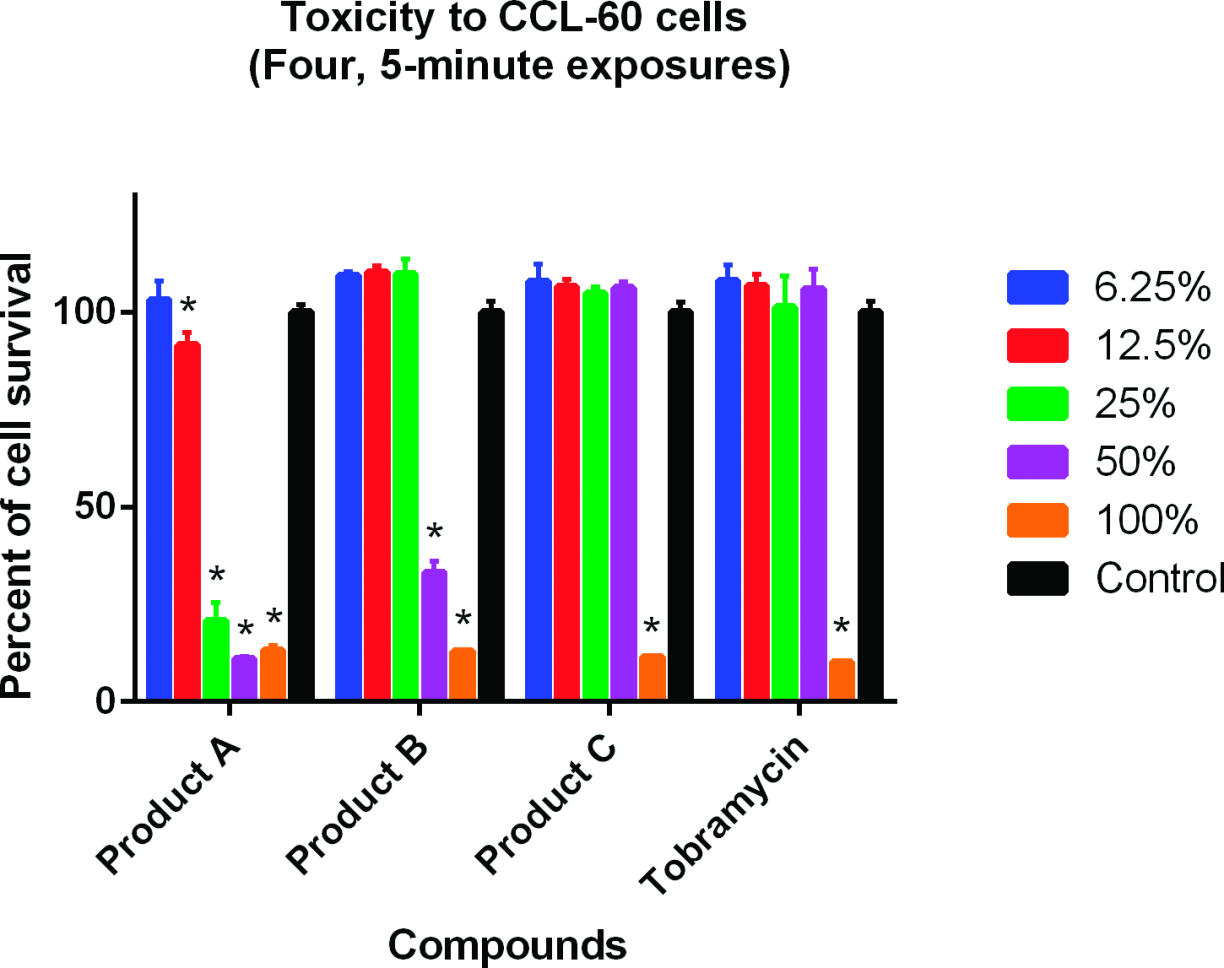
Cytotoxicity of ophthalmic products against CCL-60 cells, four 5-minute exposures. Tobramycin was used as the positive control. Cells were exposed to the commercial products for 5 minutes, 4 separate times. Cell viability was expressed as percentage of absorbance in comparison to the negative control. A compound was considered toxic to the cell line in this experiment if ≤ 95% cell survival was recorded. Toxicity is indicated by (*).

Results for the cytotoxicity analyses are shown in Figs 1–4. In the single 2-hour assay using the J774A.1 cells, both Products A and B were less toxic than tobramycin 0.3% (Fig 1). In the single 2-hour assay using the CCL-60 cells, Product B was less toxic than Product A and tobramycin 0.3% (Fig 2). In the four 5-minute interval exposure assay using the J774A.1 cells, all three products (A, B and tobramycin 0.3%) exhibited similar toxicities at dilutions of 6.25% and greater (Fig 3). In the four 5-minute interval exposure assay using the CCL-60 cells, products A and B were more toxic than tobramycin 0.3% exhibiting toxicities at dilutions of 50% or greater (Fig 4). In both of the single 2-hour exposures using the J774A.1 cells and the CCL-60, Product C exhibited no toxicity at dilutions < 100% (Figs 1 and 2). For the four-5 minute interval exposures using the J774A.1 cells and CCL-60 cells, Product C exhibited no toxicity at dilutions ≥ 50% (Fig 3) and no toxicity at dilutions ≥ 100% (Fig 4), respectively.

## Discussion

In this model Products A and B showed an inhibition of growth similar to and slightly less than the positive controls, while Product C was unable to inhibit growth of any of the bacteria common to the ocular surface. Products A and B exhibited toxicities similar to the positive controls, while Product C exhibited minimal toxicity. These products were chosen because of their commercial availability in the veterinary market.

Previous studies have demonstrated that CSA-13, as a raw compound, was very active against both Gram-positive and Gram-negative bacteria. Chin et al. measured the MIC of CSA-13 against clinical isolates of *S*. *aureus* [25]. The MIC values achieved in that study ranged from 1–2 μg/ml. In the present study, *S*. *aureus* was inhibited at a concentration of 6.25 μg/ml by Products A and B, a higher MIC than was achieved in the Chin et al. study. Moscoso et al. evaluated the inhibitory activity of CSA-13 against various *Streptococcus* spp. [48]. MIC results against these Gram-positive strains ranged from 1–8 μg/ml. In the present study, Products A and B were inhibitory against *Streptococcus canis* at concentrations between 6.25–12.5 μg/ml, again slightly higher than what was achieved in the comparison Moscoso et al., study. The higher MIC values of *Streptococcus canis* as compared to the *Staphylococcus* spp. may be due to the interaction with serum components in the culture media used with the *Streptococcus* spp. In the Moscoso study, MHB was supplemented with 2.5% horse blood, and in the present study, MHB was supplemented with 5% sheep’s blood. The interaction with serum components has been shown to reduce the effective concentration of CSA-13 in blood-product supplemented media [48].

In 2008, Chin et al. evaluated CSA-13 against clinical isolates of *Pseudomonas aeruginosa*. The MIC_50_ of the fifty clinical isolates in that study was 16 μg/ml [24]. The MIC of Products A and B against the *Pseudomonas aeruginosa* isolates in the current study ranged from 6.25–25 μg/ml. Interestingly, Chin et al. also evaluated tobramycin and ciprofloxacin against the same clinical isolates and achieved MIC_50_ values of 1 and 2 μg/ml, respectively [24]. In the present study, the MIC of tobramycin was ≤ 0.36 and ranged for tobramycin between ≤ 0.36 and 5.8 μg/ml. Similar to the Chin study, CSA-13 in the present study was less inhibitory than tobramycin, but similar in inhibition to the ofloxacin. The disparity in the MICs of the clinical *Pseudomonas aeruginosa* isolates between the Chin, et al. study and the present study could be related to the pathogenicity of the isolates used and/or the number of the isolates used.

The active ingredient in Product C is neutral SOW. However according to Sonoma Pharmaceuticals, the patented Microcyn^®^ technology is no longer used to produce Product C (as indicated on their webpage). Two studies have evaluated the antimicrobial activity of neutral SOW. However, those studies used the patented Microcyn^®^ technology when producing the active ingredient. To the authors’ knowledge, there are no studies that have evaluated the antimicrobial activity of the neutral SOW used in the current Product C. In 2005, Landa-Solis et al. showed that a stable SOW (Microcyn^®^) with a low oxychlorine content (51–85 ppm) and neutral pH was able to kill *S*. *aureus* and *P*. *aeruginosa* within 20 seconds [38]. In 2010, Sauer et al. demonstrated that exposure of *P*. *aeruginosa* to neutral SOW (Microcyn^®^) for 20 seconds (during exponential phase) with oxychlorine concentrations of 80, 125 and 200 ppm were sufficient to reduce viability by more than 5 logs. Further, exposure for 10 minutes to neutral SOW with oxychlorine concentrations of 125 and 200 ppm was sufficient to eradicate stationary phase *P*. *aeruginosa* [32]. In the present study, Product C was unable to inhibit growth of any of the bacterial organisms tested over a 24-hour period and a free available chlorine or oxychlorine concentration of 150 ppm. While the time-kill assay used in the Landa-Solis and Sauer studies differed from the assay (MIC) used in the present study to measure inhibition of bacterial growth, there is a stark difference in growth inhibition between the studies. Perhaps the different method for producing the SOW is responsible for this difference.

The cytotoxic effects of Products A and B were evaluated against a murine macrophage-like cell line (J774.1) and a rabbit corneal fibroblast cell line (CCL-60) using the MTS assay. Results revealed toxicities similar to slightly less than the positive control, tobramycin for the J774.1 assays and similar to slightly more than tobramycin for the CCL-60 assays. Tobramycin was selected as the positive control due to the lower toxicity compared to gentamicin and ofloxacin [49, 50]. While less toxic than tobramycin, the toxicities of Products A and B may have been diminished due to an interaction with the fetal bovine serum used in the J774.1 MTS assay. As discussed previously, the interaction with serum components has been shown to reduce the effective concentration of CSAs in blood-product supplemented media [48]. Product A was more toxic than Product B and tobramycin in both CCL-60 assays. As the chemical composition of Products A and B is the same, the difference in toxicities between the two compounds may be related to stability and shelf-life as Product B was older than Product A.

Product C was only cytotoxic in the single 2-hour exposure assay when placed undiluted on both the J774.1 and CCL-60 cell cultures. In the four 5-minute exposure assays using the J774.1 assay, Product C was cytotoxic when undiluted and diluted 1:1 (50%) with the DMEM media supplemented with 10% fetal bovine serum. Product C was cytotoxic in the CCL-60 assay only when placed on cell culture undiluted. To the authors’ knowledge, there are no previous studies evaluating the effects of a neutral SOW against the J774.1 murine macrophage-like cell line or the CCL-60 rabbit corneal fibroblast cell line.

Product C is composed of water and a combination of hypochlorous acid and sodium hypochlorite, similar to a dilute bleach. A study in 2001 evaluated the shelf life of undiluted bleach and determined that undiluted bleach was able to maintain 90 per cent of its original strength for at least six months [50]. However, dilute bleach in clear syringes exposed to sunlight demonstrated a rapid loss of chlorine content [50]. The authors concluded that dilute bleach should not be stored for more than three months [51]. The Product C used in this study was purchased off of the shelf in a clear blue bottle. The length of time since the product was manufactured was unknown. Perhaps degradation of the hypochlorous acid was the reason for the diminished activity in this study.

We have demonstrated the potential utility of Products A and B against clinical isolates from the eye and their safety when placed on cell culture. The CSAs were similar to slightly less effective than common commercial antibiotics, but still demonstrated moderate to potent bactericidal activity and toxicities similar to a known, minimally toxic, ophthalmic antibiotic solution, tobramycin. However, the neutral super-oxidized water, Product C, was an ineffective antimicrobial alternative. Moving forward, Products A and B may offer alternatives or co-treatment modalities to current approaches and have the potential to be used for treatment of infections of the ocular surface caused by common bacterial flora. However further investigations are needed.

## Supporting information

S1 Table Description of clinical isolates used in the study. (DOCX)
