# Supplementary material for: Antibacterial activity and safety of commercial veterinary cationic steroid antibiotics and neutral superoxidized water
Source: PLoS One. 2018 Mar 7;13(3):e0193217. doi: 10.1371/journal.pone.0193217 (PMC5841667; doi:10.1371/journal.pone.0193217)
Supplement: S1 Table — (DOCX) [file pone.0193217.s001.docx]

**Table 1: Description of clinical isolates used in the study**

| Strain | Description | References |
| --- | --- | --- |
| *Moraxella bovis* 80-2013000742 | Clinical isolate from infectious bovine keratoconjunctivitis, , Oklahoma | ([1](#_ENREF_1)) |
| *Moraxella bovis* 36-2012001922 | Clinical isolate from infectious bovine keratoconjunctivitis, California | ([1](#_ENREF_1)) |
| *Moraxella bovis* 120-2014002878 | Clinical isolate from infectious bovine keratoconjunctivitis, North Carolina | ([1](#_ENREF_1)) |
| *Moraxella bovis* 42-2012003587 | Clinical isolate from infectious bovine keratoconjunctivitis, California | ([1](#_ENREF_1)) |
| *Neisseria spp.* isolate 1 | Clinical isolate from dog conjunctiva, Indiana | This study |
| *Pseudomonas aeruginosa* 15442 | Quality control strain for testing antimicrobial agents | ([2](#_ENREF_2)) |
| *Pseudomonas aeruginosa* isolate 1 | Clinical isolate from dog conjunctiva, Washington State | This study |
| *Pseudomonas aeruginosa* isolate 2 | Clinical isolate from dog conjunctiva, Washington State | This study |
| *Staphylococcus aureus* isolate 1 | Clinical isolate from dog conjunctiva, Indiana | This study |
| *Staphylococcus aureus* isolate 2 | Clinical isolate from dog conjunctiva, Washington State | This study |
| *Staphylococcus pseudintermedius* Case one | Clinical isolate from dog conjunctiva, Washington State | ([3](#_ENREF_3)) |
| *Staphylococcus pseudintermedius*  Case two | Clinical isolate from dog conjunctiva, Indiana | ([3](#_ENREF_3)) |
| *Streptococcus canis* isolate 1 | Clinical isolate from dog conjunctiva, Indiana | This study |
| *Streptococcus canis* isolate 2 | Clinical isolate from dog conjunctiva, Indiana | This study |
| *Streptococcus canis* isolate 3 | Clinical isolate from dog conjunctiva, Indiana | This study |
| *Streptococcus canis* isolate 4 | Clinical isolate from dog conjunctiva, Indiana | This study |
| *Streptococcus schleiferi* isolate 1 | Clinical isolate from dog conjunctiva, Indiana | This study |

1. Loy JD, Brodersen BW. Moraxella spp. isolated from field outbreaks of infectious bovine keratoconjunctivitis: a retrospective study of case submissions from 2010 to 2013. J Vet Diagn Invest. 2014; 26: 761-768.

2. Mohamed MF, Brezden A, Mohammad H, Chmielewski J, Seleem MN. A short D-enantiomeric antimicrobial peptide with potent immunomodulatory and antibiofilm activity against multidrug-resistant Pseudomonas aeruginosa and Acinetobacter baumannii. Sci Rep. 2017; 7:6953.

3. Hamed MI, McCalla TL, Townsend WM, Seleem MN. Staphylococcus pseudintermedius Isolated from two dog cases witih ophthalmic lesions. Am J Infect Dise Microbio. 2017; 5:132-136.
